# Report of Thirteen Cases of Ununited Fracture, Treated by Sub-Cutaneous Perforation of the Bone

**Published:** 1858-09

**Authors:** Daniel Brainard

**Affiliations:** Prof. of Surgery in the Medical College of Chicago, Corresponding Member of the Society of Surgery of Paris, of the Medical Society of the Canton of Geneva, Switzerland, of the Rhode Island State Medical Society, etc., etc.


THE CHICAGO

MEDICAL JOURNAL.

VOL. I. SEPTEMBER, 1858. No. 9.

### ORIGINAL COMMUNICATIONS.

ARTICLE I.

### REPORT OF THIRTEEN CASES OF UNUNITED FRACTURE, TREATED BY SUB-CUTANEOUS PERFORATION OF THE BONE.

BY DANIEL BRAINARD, M.D.,

Prof. of Surgery in the Medical College of Chicago, Corresponding Member of the Society of Surgery of Paris, of the Medical Society of the Canton of Geneva, Switzerland, of the Rhode Island State Medical Society, etc., etc.

In the spring of 1854, I had the honor of submitting to the American Medical Association an essay on the treatment of false joint and delayed union of fracture by a new method. The committee of the association awarded the annual prize to this essay. It contained, besides the experiments and reasoning on which the method is based, a report of cases treated by the method proposed.

Since the publication of that essay, a more extended experience has enabled me to form a more just appreciation of the value of the treatment proposed, to ascertain its advantages and defects, and to suggest some improvements in the manner of its performance. I think it due to the profession, as well as to myself, that the results of this practice should be fully and fairly laid before the public. It will be seen by the result of the cases herein detailed, that the views formerly expressed concerning the efficacy and safety of the operation are in the main confirmed. It may be noticed, also, that in those cases in which this operation did not succeed, others more severe also failed.

As was to be expected, several surgeons discovered that this operation was not new; that they had themselves performed it with bistouries, gimlets and other instruments quite incapable of being made to penetrate sound bone; and they have accompanied the publication of their claims to priority with comments more or less unjust.

For this reason, I hope to be pardoned for repeating here what was written in the preface of my first publication on the subject, page 8: "When I speak of novelty, let no one impute to me the pretension to have been the first to attempt to institute a similar treatment. On the contrary, I recognize with pride that many surgeons have before me attempted the same object." Among these I cited at different times: Sir Charles Bell, who suggested but did not perform it; Blandin, who used a bistoury, and whose patient is said to have died of hemorrhage; Guerin, who has done so much for sub-cutaneous surgery, and who filed the ends of the bones; Sanford, of Iowa, who came nearer to my method than any other surgeon, and Miller. Of all these attempts I was in ignorance when I first performed my operation, but became aware of them before making my experiments public. The profession was equally in ignorance of them. At the present time, not one of these methods is, as far as I can learn, resorted to. It is, therefore, not too much to claim, that the instrument, the method of using it, and the principle upon which it acts, are of my own discovery.

### I. UNUNITED FRACTURE OF THE HUMERUS.

Four of the cases which have fallen under my care were ununited fractures of the humerus. As these are among the most intractable of all, they are therefore the severest tests of the value of any method of treatment.

CASE 1. Ununited fracture of the humerus, of four months' standing. Two perforations of the bone. Cure in one month.

P. P., a mechanic, of good constitution, aged thirty years, received, October 3d, 1856, an oblique fracture of the humerus, immediately below the attachment of the tendon of the pectoralis major muscle. The injury was severe, and although the fracture was not compound, there was great contusion of the member, and a wound existed behind the olecranon process which extended to the bone. Dressing performed with great care in the usual manner, a pad being placed between the arm and thorax.

Everything progressed favorably, and, at the end of three weeks, the wound of the elbow was cicatrized, and considerable firmness in the fracture, which was in perfect apposition. At this time, the patient, who was a carpenter, and employed as a watchman about a building, fell about six feet, breaking up all adhesion, and displacing the fragments. The dressings were readjusted, and at the end of about four weeks more, the union had taken place. At this time, he received another injury, refracturing the bones and displacing the fragments.

From this time, there was no tendency to union; the upper fragments projected, and although the arm by careful measurement was one-eighth of an inch longer than the other, the apposition of the fragments was with difficulty and somewhat imperfectly maintained.

The treatment was, however, persevered in until 13th Feb., 1856, over four months after the first accident, when, as there was no tendency to union, Drs. N. S. Davis, W. B. Herrick, J. W. Freer and C. Duck, were asked to give their advice in the case. They recommended the sub-cutaneous perforation of the bone, which was done in their presence, in the following manner: With a medium-sized perforator I penetrated and traversed the fragments in three different directions, viz: horizontally, obliquely upwards and obliquely downwards. The dressings were applied with great care.

*Feb. 23d.* Dressings removed, and there being no tenderness at the seat of fracture, the operation was repeated with a larger-sized instrument, and the wounding of the adjacent surfaces of the fracture effected more extensively. The dressing was reapplied with great care. On the 30th, union was found to have taken place.

CASE 2. Ununited fracture of the humerus, of eight months' standing. Four operations by perforation.

H. C. P., aged twenty-nine years, of good constitution, a resident of Nebraska Territory, received, in Sept., 1855, a gun-shot wound from a large musket ball, which fractured and extensively comminuted the left humerus about the middle, lacerating the soft parts extensively. The ball entered the fore part of the arm, and passed out behind. The swelling and tenderness were such, that splints were at first very imperfectly applied. Several abscesses formed about the point of fracture and as high up as the shoulder, and fragments of bone were discharged from time to time.

He presented himself to me, April 16th, 1856, more than seven months after the accident. There was no tendency to union. A fistulous opening existed at the point of entrance of the ball, from which healthy pus was discharged. On passing a probe through this opening, numerous fragments of bone were met with, lying between the fractured extremities.

Having enlarged this opening with the bistoury, I removed four of the fragments. They were of large size, and composed of the compact structure of the bone. Two of them were each over an inch in length. The usual dressing, composed of four splints of wood, were then applied, and continued with care for eight weeks. At the end of this time, the wound had cicatrized, but there was no tendency to union. The loss of bony substance was in this case so great, that there was no overlapping, but the extremities of the fragments were rounded and lay nearly in contact with each other.

*June* 1*st*, 1857. I passed down upon the point of fracture a large sized perforator, and directing it first upward, penetrated the upper fragment deeply at two different points, then separated, by scratching, its fibrous covering. This treatment was repeated upon the lower fragment without withdrawing the instrument, but directing its point downward. It was then withdrawn, a piece of adhesive plaster applied on the point of puncture, and the splint and bandages reapplied as before.

This operation was repeated three times, at intervals of ten days. After the second puncture, a commencement of union was perceptible, and he left, August 31, 1856, to attend to some business at a distance. At this time, the arm was examined by Dr. J. C. Morfit and others, and union found far advanced.

Since writing the above, I have learned that the patient went under the care of another surgeon, and had a seton inserted without benefit.

CASE 3. Ununited fracture of the humerus, of six months' standing. Five operations by perforation. Cure in six weeks.

C. N., aged twenty-four years, a young man of good health, received, June 2d, 1855, a simple fracture of the lower third of the right humerus, by a fall from a carriage. He states that it was dressed with pasteboard splints only four inches in length confined by a roller. This treatment was continued about three months, after which it was left entirely without dressing.

*Dec. 12th*, 1855. The fracture could be distinctly felt to be very oblique, but not much overlapped. The bone bent freely in every direction, but the fractured surfaces did not glide upon each other. I perforated the extremities of the fragments in two different directions where they overlapped, and applied the splints of wood as usual. This treatment was repeated four times, at intervals of one week. On removing the dressings, Jan. 28th, 1856, union was found to have taken place. The splints were, however, continued a week longer, when they were removed.

CASE 4. Ununited fracture of the humerus, of five months' standing. Treatment for five weeks by six perforations. Use of seton and resection without benefit.

Patrick Gavin, of Waukegan, Ill., received, Nov. 20th, 1856, a simple fracture of the left humerus, below the middle, and at the same time a fracture of the left tibia.

The latter fracture united at the usual time, but the former, Feb. 26th, 1857, showed no tendency to union. The tissues of the arm were flabby, and the ends of the fragments glided freely in any direction.

Perforated the ends of the fragments freely, and applied splints, with the forearm extended, this being the only position in which immobility could be preserved.

*March 10th*. Removed splints, and repeated the operation.

*March* 25*th*. Repeated the operation; much less mobility.

*April* 5*th*. Repeated operation; considerable firmness.

*April* 13*th*. Repeated operation; movements very slight.

*Aug*. 6*th*. A seton was placed between the ends of the fractured bone this morning. The seton consisted of four threads of cotton.

*Aug*. 7*th*. Some pain and swelling.

*Aug*. 8*th*. Suppuration commenced around the seton.

*Aug*. 11*th*. The seton was removed to-day. The patient has suffered much pain and swelling from its introduction. The inflammation is very severe. Splints were carefully applied, and continued for four months. Fistulous openings remained for two months. No tendency to union.

*Feb*. 6*th*, 1858. A vertical incision having been made down to the ends of the bone, they were removed to the extent of one-fourth of an inch from each extremity. A dressing was applied, consisting of splints placed one upon the internal, anterior and posterior sides of the arm with rollers. The wound was filled with lint, in order that it might heal from the bottom.

*Feb*. 7*th*. Very severe symptoms, both local and constitutional, followed the operation. The arm was much swollen and very painful. The patient was affected with nausea and vomiting. The dressing upon the arm was loosened and an evaporating lotion applied. Quiniæ sulph. and morphiæ sulph. were administered internally, together with broths.

*Feb*. 8*th*. Patient somewhat better this morning. Continued same treatment.

*Feb*. 10*th*. Patient better. The swelling in the arm having somewhat subsided, the dressing was removed and carefully reapplied. The wound has commenced to suppurate. An opening was left in the dressing so that the wound might be dressed each day.

*Feb*. 16*th*. The dressing was removed this morning; wound granulating; no tendency to union.

*Feb*. 20*th*. Arm examined; no tendency to union.

*March* 1*st*. The wound made in the arm has healed. The fracture still movable. Four splints were applied this morning, one upon each side of the arm, extending from the shoulder to the elbow, with a starch bandage.

*March 15th.* Dressing removed; no union. Dressing carefully reapplied.

*April 10th.* Arm examined again this morning; no *union.*

*May 10th.* Two sheet-iron splints made to fit the arm were applied, which the patient still wears, no union having taken place.

II. FIVE CASES OF UNUNITED FRACTURE OF THE FEMUR.

CASE 5. Ununited fracture of the right femur of five months standing. Treatment by eleven perforations during five months. Cure.

Frederick Gaylord, of Wisconsin, aged thirty-five years, of delicate health, received, January 17th, 1855, an oblique compound fracture of the right femur, a little above the middle. This, according to his account, was dressed by the application of short wooden splints around the thigh, secured by many turns of a roller applied very tightly. No extension was used, and no roller upon the leg. This treatment was continued for five weeks. When the splints were taken off, there was great excoriation of the knee from pressure. Œdema of the leg and foot. The opening in the skin was healed.

He entered the hospital of the "Sisters of Mercy," at Chicago, April 25th, 1855, under the care of Dr. Johnson, the surgeon in attendance at that time.

*June 1st*, 1855. Came under my care. The member was found shortened about two and a half inches, notwithstanding Dr. Johnson had applied suitable splints with extension and counter-extension. The whole member œdematous. Cicatrices about the knee, and one on the anterior part of the thigh, from the protrusion of the upper fragment lying in front of the lower, and much overlapped. No commencement of union, but free gliding and bending. Patient's health poor, emaciation, pallor of the surface, no appetite.

I dressed the member with much care in the straight position, using the dressing of Dessault, perforating the bone from before backwards in three different directions. Directed a nourishing diet.

In boring the bones, it was noticed that when the perforator had passed through one fragment, it slipped about a quarter of an inch before touching the other.

*June 13th.* Repeated the same operation at a different point. Fragments seemed half an inch apart at the point perforated.

*June 24th.* Repeated the operation.

*June 29th.* Repeated the operation. Inadvertently, the puncture was made on the cicatrix, and four days afterwards it was found that an abscess had formed at that point. This was opened, and as the pus was serous, the cavity was washed out with sul. copper, four grains to the ounce of water.

*July 27th.* Abscess quite healed.

*Aug. 24th.* In order to avoid the vicinity of the abscess, the perforation was made from the outside of the thigh; the instrument then passed between the fragments, which seemed not to touch each other at any point. Denudation of the surfaces was effected to as great an extent as possible.

At this time, I first learned that the patient had been in the habit of discharging with the urine a large quantity of phosphatic gravel; nitro-muriatic acid was prescribed.

*Sept. 8th.* Repeated perforation from the outside in the same manner.

*Sept. 28th.* Perforated from the anterior surface.

*Oct. 18th.* Repeated last operation.

*Nov. 3d.* I used a larger sized perforator, and pressed it five times through the bone without withdrawing it from the skin. Space between the bones evidently becoming filled with more solid substance. This was the last operation. There was much firmness.

*Dec. 18th.* Removed all the dressings; union perfect.

This case presented such a combination of unfavorable circumstances as to render it one of the most difficult I have met with.

I saw Mr. Gaylord, in Jan., 1858, in good health, with a good and useful limb.

CASE 6. Ununited fracture of the femur, of four months' standing. Four operations. Cure in six weeks.

Peter Mullen, aged fifty-six years, entered the Mercy Hospital for a simple oblique fracture of the right femur. It was dressed in the usual manner. Soon after, he fell into a severe paroxysm of epilepsy, which continued over an hour, and displaced the dressings. .These were readjusted without delay, but an attack of typhoid fever supervened, which lasted about four weeks. He then convalesced slowly.

At the end of sixteen weeks, there was not the slightest commencement of union. At this time, Oct. 20th, 1854, I perforated the extremities of the fragments and reapplied the dressings.

*Oct. 29th.* Repeated the operation, using a large sized instrument, and passing it in laterally.

*Nov. 10th.* Repeated the operation; considerable firmness.

*Nov. 18th.* Repeated perforation; union greatly advanced; tenderness at the seat of fracture.

*Dec. 1st.* Union perfect.

CASE 7. Ununited fracture of the femur, of four months' standing. Cure by four perforations.

A healthy young man entered the Mercy Hospital in July, 1854, with a simple fracture of the left femur. I have not been able to obtain the notes of this case, which was under the care of Dr. Johnson, one of the surgeons of the Hospital. He came under my care in October, when I found he was being treated by my method. After four months of treatment by the straight apparatus of the Hospital, during which there was no union, Dr. Johnson bored the bones at three different times, and, Nov. 1st, there was considerable firmness, which increased till perfect union was effected.

CASE 8. Ununited fracture of the femur, of five months' standing. Cure in four weeks by one operation.

Daniel Sullivan, aged thirty-six years, a laborer, of good constitution, placed himself under treatment for an ununited fracture of the left femur, at the junction of the upper with the middle third, June 19th, 1858.

*History.*—Five months previous, viz: on the 26th day of January, 1858, he was thrown suddenly and violently from the railroad track, together with a hand-car upon which he was riding at the time, down an embankment, and by the fall received a fracture of the femur, besides several other bruises upon different parts of his body. The limb was dressed in the straight position, and allowed to remain thirteen weeks, when the dressing was removed, the attending surgeons supposing the fracture had united. In one week after the removal of the splints, the member was examined, and the fracture found to be movable. The patient at this time came under the care of Drs. White and Lake of Iowa City, who applied a starch bandage, and allowed it to remain five weeks, when it was removed. The fracture was still ununited. The limb was then dressed in the straight position, and allowed to remain two weeks, when he came to Chicago by railroad, a distance of over two hundred miles.

*Present State.*—The fractured extremities move readily upon each other. Quite a degree of angularity exists outwardly, with one and a half inches shortening. The muscles of the limb are somewhat atrophied. The patient is in good health.

*Treatment.—June* 20*th*, 1858. A perforator was passed in three different directions between and through the ends of the bone, and the limb was dressed with Dessault's splint. The patient was directed to keep as quiet as possible.

*June* 22*d*. The patient complains of some soreness in the part. Says it is a "burning pain."

*June* 27*th*. The splint was removed, and the limb examined; a good degree of firmness perceptible. Still complains of some pain, especially upon pressure or motion. Splint reapplied.

*July* 4*th*. The limb examined again this morning; improvement very perceptible since the last examination.

*July* 11*th*. Improvement still going on.

*July* 18*th*. The limb was examined; found to be united. The dressing has not been removed as yet, in order to guard against accident.

*July* 31*st*. Dressings removed; consolidation perfect.

### III. THREE CASES OF UNUNITED FRACTURE OF THE TIBIA.

All experience bears testimony to the greater facility of treating fractures of the tibia and fibula, and of the radius and ulna, by ordinary means, than of the femur and humerus; yet, while these latter may be considered the severest tests of any method of treatment, the former are not without great importance, and have peculiarities which render it necessary to study them in detail.

Case 9. Ununited fracture of the tibia, of four months' standing. Cured in two weeks by one perforation.

—— Casey, a laborer, entered the Mercy Hospital about the end of August, 1858, for an ununited fracture of the left tibia, of over four months' standing. He had been under the care of Dr. Charles Duck, and seemed to have been treated with great caution and skill, as the apposition was perfect and the movements but slight. As both the Doctor and the patient himself were decidedly of the opinion that the union was less firm than it had been a month previously, I passed a perforator in three different directions through the ends of the bones, and applied a carved splint with a foot-piece. At the end of two weeks the consolidation was perfect.

Although the slight movements which existed in this case, and the rapidity of the union, favor the supposition that it might have occurred without any operation, yet, considering the good treatment which it had received, and that the firmness was less than it had been, there seems no probability that it could have occurred without very great delay.

Case 10. Ununited fracture of the tibia, of five months' standing. Cured in five weeks by four perforations.

D. B. French, of Wisconsin, received, May 9, 1856, a double comminuted fracture of both bones of the leg. He was twenty-five years of age, and of good constitution. The upper fracture was seated four inches below the knee, and the lower four inches above the tarso-metatarsal articulation. The fracture had been extremely well adjusted immediately after its occurrence, and a union obtained of both bones at the upper fracture and of the fibula below. The tibia, however, did not unite at that point, notwithstanding the care bestowed upon him by Dr. Gordon, under whose treatment he was at the time.

*Oct. 20th.* More than five months after the accident, he came under my care. A perforator was passed in three different directions through and between the ends of the fragments; a carved wooden splint with a foot-piece was applied to the back part of the leg, and carefully secured by a roller.

*Oct. 29th.* Bored the ends of the bones and passed the instrument freely between them.

*Nov. 5th.* Repeated operation; considerable tenderness.

*Nov. 25th.* Removed dressings; consolidation complete.

CASE 11. Ununited fracture of the tibia, of seven months' standing. Cured in four weeks by four perforations.

Edwin Porter, of Wisconsin, aged twenty-one years, of good constitution, received, August 13th, 1856, a simple fracture of both bones of the right leg, a little above the middle. It appears to have been well adjusted, but, March 31st, 1857, the tibia was still ununited. The treatment was in every respect the same as in the preceding case, and in four weeks, no movements were perceptible.

IV. TWO CASES OF UNUNITED FRACTURE OF THE ULNA.

CASE 12. Ununited fracture of the ulna, of over three months' standing. Two perforations; cure in twenty-two days.

Patrick Madden, a laborer, was admitted into the Mercy Hospital, April 3d, 1854, on account of very extensive wounds received from pieces of rock, projected by the explosion of a blast. This case came under my care, May 10th succeeding. The laceration of the soft parts had prevented the coaptation of the fracture; the upper fragment projected beneath the skin, and did not touch the other. I applied splints in such a manner as to press the upper piece into its place, and retained them for nineteen days. Finding at the end of that time that there was no commencement of union, and that the pressure of the splints was painful, I perforated the bones transversely, May 27th, near twelve weeks after the fracture. Dressings replaced.

*June 8th.* Repeated the puncture, perforating the bone in three different directions.

*June 18th.* Removed the dressings; union perfect.

CASE 13. Ununited fracture of the ulna, of eighteen weeks' standing. Two perforations. Cure in four weeks.

P. M., aged thirty-five years, received, May 16th, 1854, a fracture of the left ulna, with dislocation of the radius forward. The fracture had been produced by a blow of a piece of wood, and extended into the sigmoid cavity and downward about two inches.

The subject of this accident was a drunkard of the lowest class, and the injury was not treated in any manner for about four weeks. At the end of that time, an angular splint was applied on the foreside of the member, and the bones pressed somewhat into their places, and roller applied. This treatment was continued for five weeks, and union seemed to have taken place, when the patient fell and reproduced the fracture.

He was then without any treatment until Oct. 1st, 1854. At that time there was no union. The lower fragment was not in contact with the upper, and the head of the radius was partly removed from the articular surface of the humerus.

At this date, I perforated the fragments repeatedly with the small sized instrument, and applied angular splints on the palmar surface of the member.

Considerable tenderness resulted from this operation, and some swelling.

*Oct. 12th.* The tenderness and swelling having subsided, repeated the operation.

*Oct. 28th.* Removed the dressing; union seemed perfect.

*Nov. 15th.* No movements perceptible at the point of fracture.

*Reflections.*—It will be noticed that in all the above cases no serious accident occurred. In one, a small abscess, and in another, a subject of bad constitution, some swelling, resembling erysipelas, which, however, soon subsided. These were the most serious results of about sixty perforations. We may therefore assert, with great certainty, that this operation, unless performed upon patients in a condition unfit for any operation, is entirely safe.

It will also be noticed, that, while in cases of fracture of the tibia, where apposition is perfect and the movement slight, a single perforation speedily induced union in a few days; on the other hand, fractures of the humerus and of the femur did not, in most cases, require less than four operations, nor unite in less than four weeks, while one required five months and eleven perforations to effect a cure, and another did not unite at all.

My practice at present is to commence the treatment by two or three perforations of the bone through a single opening of the skin, using an instrument of small size, repeating this every ten days or two weeks, gradually increasing the size of the instrument and the extent of the wound of the bone, until tenderness and some swelling are induced. I have very uniformly found that when pain and throbbing are felt in the seat of fracture, union has commenced.

*Direction of the Perforator.*—That point and direction of puncture should be chosen which affords the easiest access to the bony surfaces with least exposure of vessels. In many cases of oblique fracture, traversing the bones answers well. In others, as of the tibia, I have found that following the direction of fracture is best. In others, still, when the ends are not perfectly in contact, I make a perforation between, and direct the instrument first in one direction, then in the other; while in others, still, the instrument can be passed most readily between the bones and attack them at the side.

*Size of the Instrument.*—In cases of ununited fracture of the tibia, or radius and ulna, where the ends are in contact and the wounds slight, I use a perforator no more than two or three *millimetres* in breadth; while, in old cases, situated in the femur and humerus, and when there is great mobility, it is as well to use an instrument one-eighth of an inch and over in breadth. In such cases, very extensive wounding and perfect denudation is required. It was not found that the bones had in any case lost their natural feeling of density.

*Causes of Want of Union.*—In all the above cases, the causes of non-union were imperfect apposition, or a dressing admitting of too great mobility, or accidents producing displacements, or indocility of the patient. In three of the cases, the fragments were separated from each other by a sensible space, as shown by the instrument in perforating them. It is especially to be noticed, that the most efficient means for securing immobility were in every case conjoined with the treatment by perforation. These means were such as are generally known and used.

